# CT_max_ is repeatable and doesn’t reduce growth in zebrafish

**DOI:** 10.1038/s41598-018-25593-4

**Published:** 2018-05-08

**Authors:** Rachael Morgan, Mette H. Finnøen, Fredrik Jutfelt

**Affiliations:** Department of Biology, Norwegian University of Science and Technology, Trondheim, Norway

## Abstract

Critical thermal maximum (CT_max_) is a commonly and increasingly used measure of an animal’s upper thermal tolerance limit. However, it is unknown how consistent CT_max_ is within an individual, and how physiologically taxing such experiments are. We addressed this by estimating the repeatability of CT_max_ in zebrafish, and measured how growth and survival were affected by multiple trials. The repeatability of CT_max_ over four trials was 0.22 (0.07–0.43). However, CT_max_ increased from the first to the second trial, likely because of thermal acclimation triggered by the heat shock. After this initial acclimation response individuals became more consistent in their CT_max_, reflected in a higher repeatability measure of 0.45 (0.28–0.65) for trials 2–4. We found a high innate thermal tolerance led to a lower acclimation response, whereas a high acclimation response was present in individuals that displayed a low initial CT_max_. This could indicate that different strategies for thermal tolerance (i.e. plasticity vs. high innate tolerance) can co-exist in a population. Additionally, repeated CT_max_ trials had no effect on growth, and survival was high (99%). This validates the method and, combined with the relatively high repeatability, highlights the relevance of CT_max_ for continued use as a metric for acute thermal tolerance.

## Introduction

Climate change is causing an increase in global water temperatures as well as more frequent and extreme events such as heat waves^1–3^. Because the body temperature of most aquatic ectotherms conforms to that of the surrounding water, temperature is one of the main environmental factors affecting their performance, fitness and distribution^4,5^. Climate change will increase the frequency of ectothermic organisms’ exposure to transient warm events and temperatures above their tolerance range^5,6^. Therefore, understanding how aquatic ectotherms will respond to, and perform under, these conditions is driving research into thermal biology^7–10^ and, in particular, research focusing on organisms’ thermal tolerance^11–14^. Critical thermal maximum (CT_max_) is a commonly used method for measuring an animal’s upper thermal tolerance limit, and can be broadly defined as the temperature at which locomotion becomes disorganised and performance is lost during acute thermal ramping^15–18^. Through a review of papers on Google Scholar we found that the popularity for using the CT_max_ method has rapidly increased in the last decade with five times more publications using it per year between 2010–2017 than from 1990–2000.

Thermal tolerance has been suggested to have a low potential for rapid evolution^19,20^ and, based on biogeographical distribution patterns, appears relatively conserved^21,22^. Genetic variation is a requirement for adaptation^23^, and this can be estimated by calculating the heritability of a trait. Heritability (h^2^) of thermal tolerance in fish has only been estimated in a limited number of previous studies; Doyle *et al*.^24^ (h^2^ = 0.2), Meffe *et al*.^25^ (h^2^ = 0.32) and Baer & Travis^26^ (h^2^ = 0.15). In addition, technical complications in some of the selection lines make interpretation of Baer and Travis’^26^ results challenging, limiting the useful number of studies on heritability further. Therefore, more data on the heritability of thermal traits are needed for robust estimates to be made^7^.

Another approach for estimating adaptation potential of thermal tolerance is to assess its repeatability, due to the positive relationship between repeatability and heritability^27^. The repeatability of a trait sets an upper limit on its heritability, as it includes both genetic and environmental variance^27^. By quantifying the proportion of total variation of a trait that is due to differences between individuals, repeatability shows the consistency of an individual’s performance over a longer time scale^28,29^. If thermal tolerance is inconsistent within an individual over time (i.e., low repeatability), then the adaptive potential of the trait is low^30^. Despite the apparent need to estimate the repeatability of thermal tolerance there has been no study, to our knowledge, that has investigated this in aquatic ectotherms.

Although there are many studies that use CT_max_, very few report survival after a CT_max_ test. Of the studies that do, survival is claimed to be above 90%^31–33^. In zebrafish however, only one study^34^ has looked at survival after CT_max_ and although they had high survival in fish acclimated to 20 °C, a low survival rate was seen in fish acclimated to 30 °C. This suggests that CT_max_ tests can have negative physiological consequences for fish, which may be under-reported in the literature.

The aim of this study was to determine whether CT_max_ is a repeatable trait within an aquatic ectotherm, the zebrafish (*Danio rerio*), and thereby establish its potential applicability as a metric for thermal tolerance. In addition, we aimed to validate the CT_max_ method, which is widely used in the field of thermal biology, by testing whether repeated CT_max_ tests affect zebrafish growth and survival. We hypothesized that CT_max_ is a repeatable trait, that multiple CT_max_ tests negatively affect zebrafish growth, and that survival after CT_max_ tests would be high.

## Results

### Repeatability of CT_max_

**Figure 1:**
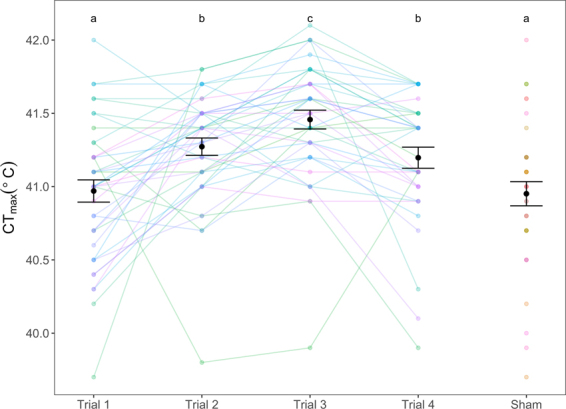
Critical thermal maxima, CT_max_(°C), for zebrafish (n = 40) repeatedly measured over four trials one week apart, as well as for the sham fish (n = 38) whose CT_max_ was recorded simultaneously as Trial 4. CT_max_ was measured at a thermal ramping rate of 0.3 °C min^−1^. Letters a, b & c illustrate statistically significant differences between trials. Coloured points and lines represent individual fish’s CT_max_. Black points and bars show the mean CT_max_ ± s.e.m. for each trial.

The repeatability of CT_max_ was estimated as 0.216 (95% C.I.: 0.066–0.434) when all CT_max_ trials were included and increased to 0.447 (95% C.I.: 0.284–0.645) when the first CT_max_ trial was omitted from the model. CT_max_ also increased from the first (40.97 ± 0.10 °C) to the second trial (+0.30 ± 0.10 °C, F_4,125_ = 10.98, p < 0.01, Fig. 1). In the third trial CT_max_ increased further, (+0.19 ± 0.08 °C, p = 0.03) before declining in the final trial (−0.26 ± 0.08 °C, p = 0.003) to the level of trial 2 (F_4,125_ = 10.98, Fig. 1).

### Acclimation response

**Figure 2:**
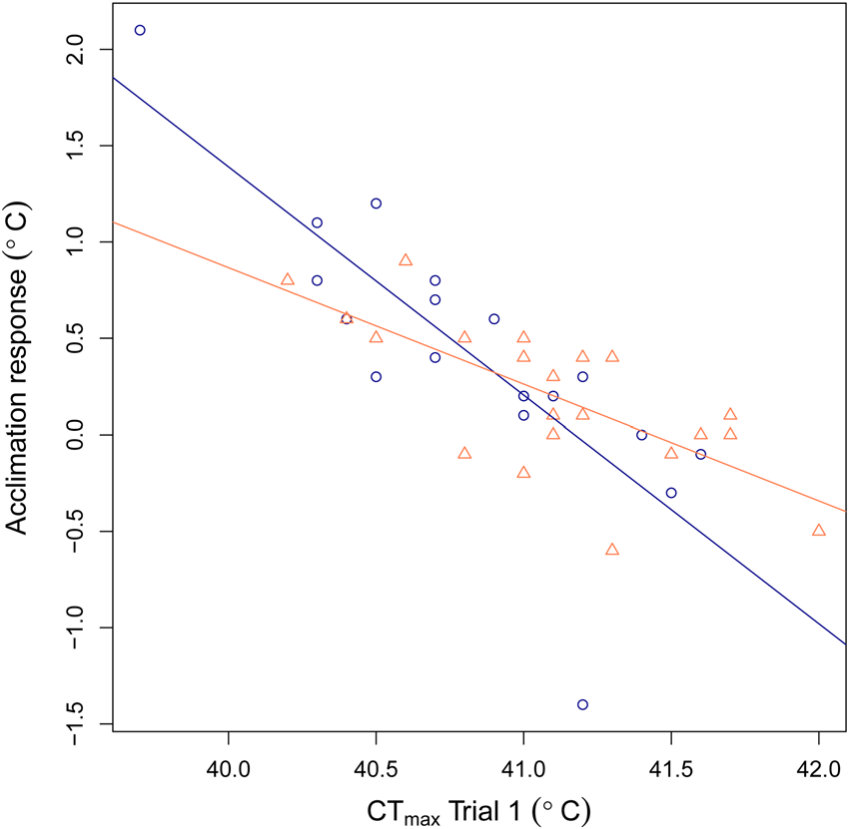
The relationship between an individual fish’s initial or innate CT_max_ (Trial 1) and their acclimation response (change in CT_max_ from Trial 1 to Trial 2) (n = 40). There was a one week interval between Trial’s 1 and 2 and a thermal ramping of 0.3 °C min^−1^ was used to determine CT_max_. Females are represented by orange triangles and an orange regression line and males by blue circles and a blue regression line.

A negative relationship was found between an individual’s initial CT_max_ and their acclimation response; the change in CT_max_ from trials 1 to 2 (F = 53.92_1,36_, p < 0.01, Fig. 2). Therefore, individuals with a low CT_max_ in trial 1 had a larger increase in their score from trial 1 to trial 2 than individuals with a high initial CT_max_ (Fig. 2). This relationship appeared more pronounced in males than in females (Sex × CT_max_Trial1, F = 5.35_1,36_, p = 0.03), however since this interaction is driven by two male outliers it is unclear if this has true biological meaning. The correlation between an individual’s CT_max_ in trial 1 and trial 2 was therefore weak (r = 0.15, p = 0.35).

### Effect of CT_max_ on growth

**Figure 3:**
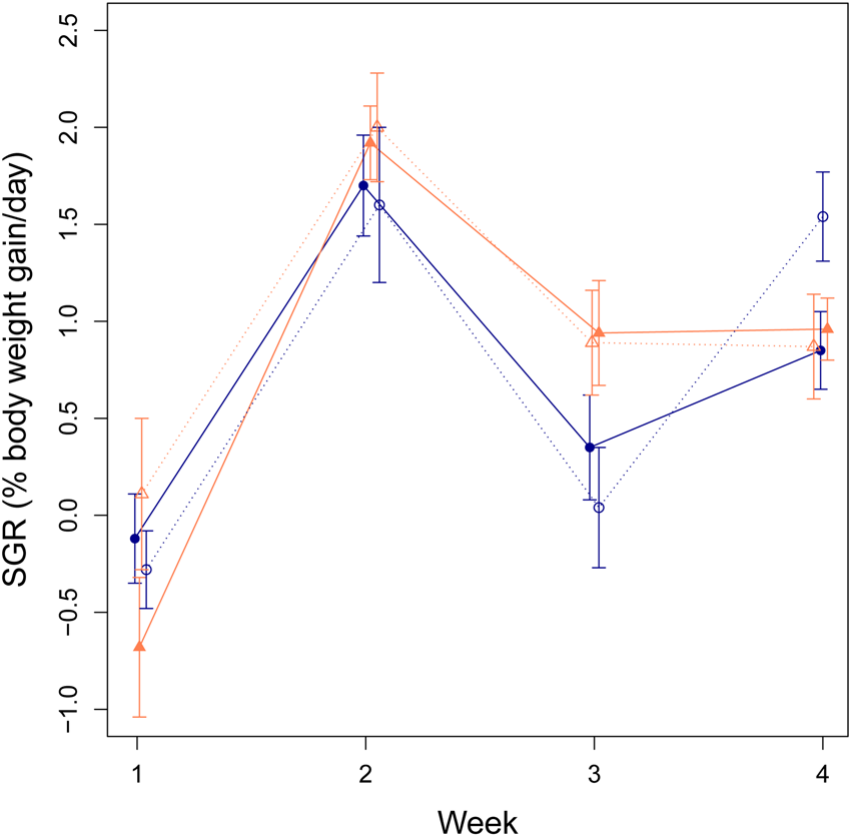
Specific growth rate (SGR) (% body weight gained/ day) over four time intervals, from tagging until the first CT_max_ test (Week 1) and then weekly thereafter (Weeks 2–4). Solid lines and filled symbols represent the repeatability group (which underwent weekly CT_max_ tests) and dashed lines and open symbols represent the sham group (which only had one CT_max_ test in Week 4 and experienced sham CT_max_ tests in Weeks 1–3). Females are represented by orange triangles and males by blue circles. Values are given as means ± s.e.m.

There was no difference in growth between the sham and repeatability groups (F_1,304_ = 1.03, p = 0.31, Fig. 3), and growth did not differ between the sexes (F_1,304_ = 0.58, p = 0.45; Fig. 3).

### Size and CT_max_

**Figure 4:**
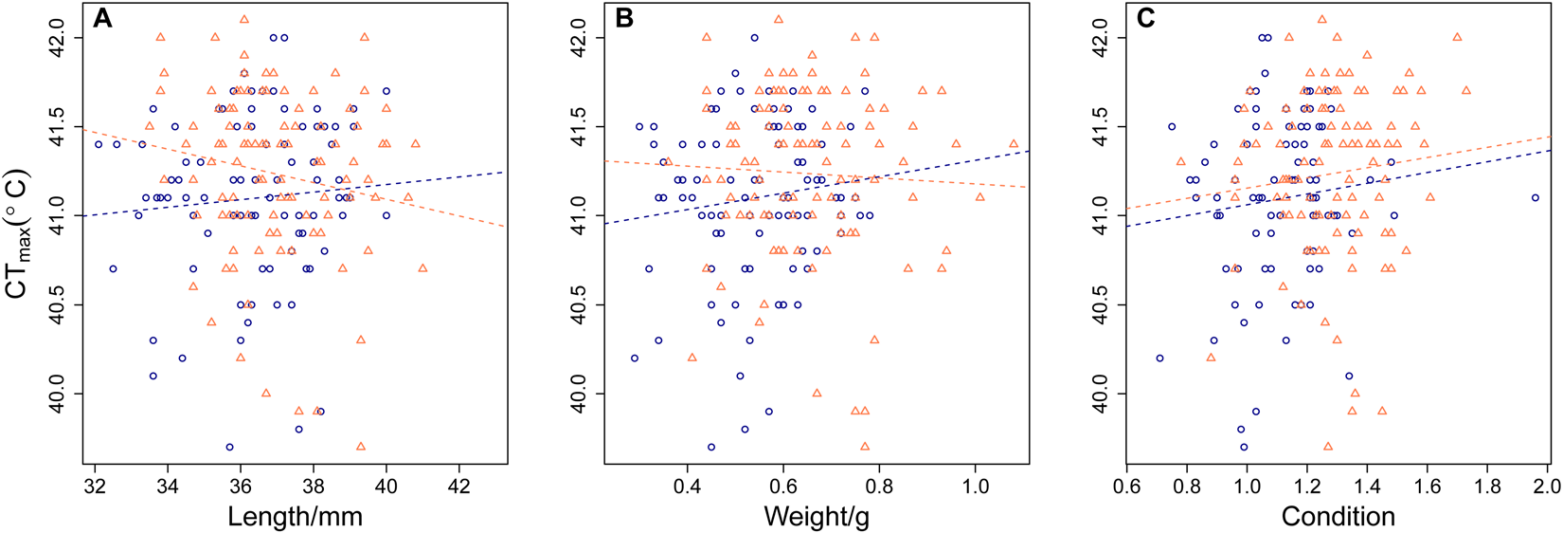
Relationship between CT_max_ and three size metrics: (**A**) Length, males: F = 0.59_1,__86_, p = 0.45, R^2^ = 0.01, females: F = 2.88_1,86_, p = 0.09, R^2^ = 0.03. (**B**) Weight, males: F = 1.22_1,88_, p = 0.29, R^2^ = 0.01, females: F = 0.24_1,102_, p = 0.62, R^2^ = 0.01. (**C**) Condition index, males: F = 1.11_1,86_, p = 0.29, R^2^ = 0.01, females: F = 1.12_1,102_, p = 0.29, R^2^ = 0.01. Females are shown by orange triangles and males by blue circles.

There was no relationship between CT_max_ and length, weight and condition (Fig. 4) and CT_max_ did not differ between the sexes (F_1,107_ = 1.31 p = 0.26, Fig. 4).

## Discussion

We show that after an initial heat shock CT_max_ is repeatable over three trials within individuals, meaning that consistent individual differences exist over medium timescales. The repeatability when all four trials were included was significant but not high. However, the repeatability increased when the first trial was excluded. This increase in repeatability could be caused by the increase in CT_max_ from the first to the second trial, where an acclimation response altered the thermal tolerance differently depending on the initial CT_max_ response.

When fish undergo a heat shock such as a CT_max_ trial, physiological mechanisms associated with warm acclimation are activated. Such mechanisms can include increased heat shock protein production^35,36^, changes in membrane fluidity^37,38^, protein isoforms^39,40^, and altered mitochondrial density^41^. Such adjustments are considered beneficial for coping with future thermal challenges^36^. Similar increases in CT_max_ have been shown in earlier studies^15,42,43^, which named the effect “heat hardening”. Due to these acclimation effects, individuals were physiologically different in the second trial than they were in the first trial. Whilst CT_max_ increased further in the third trial, it was to a lesser extent. This suggests that the biggest physiological responses occurred between the first and the second trial.

Whilst thermal tolerance increased from the first to the second CT_max_ trial at the group level, there was large individual variation. Some individuals had high innate thermal tolerance and were therefore top performers in the first trial, however, the same individuals appeared less plastic in their acclimation response and were thus not top performers in subsequent trials. Indeed, their change in CT_max_ in the second trial was minimal or negative. Conversely, individuals with a low innate thermal tolerance (i.e. the poor performers in trial one) had a large capacity for acclimation, and increased their thermal tolerance, becoming top performers in the second trial. This effect is not due to the fish reaching their maximum post-acclimation thermal tolerance, as we have measured CT_max_ of above 43 °C after longer acclimation (Morgan *et al*. unpublished data). This shows that zebrafish have varying levels of thermal plasticity and capacity for acclimation between individuals, and it can explain the increase in repeatability scores as acclimation allows the group to become more heat tolerant as a whole. These individual differences in acclimation response suggest two different tolerance strategies: (1) having a high innate thermal tolerance and a low level of thermal plasticity, or (2) having a low innate thermal tolerance and a high level of thermal plasticity.

Acclimation, or “heat hardening”, inevitably occurs between the first and second CT_max_ challenges. The first trial therefore represents the innate thermal tolerance while the second trial gives a measure of the acclimated thermal tolerance. It may be impossible to get a true estimate of the repeatability of the innate thermal tolerance, as a thermal challenge such as CT_max_ can only be experienced as novel once. An estimate of repeatability that includes the first CT_max_ may therefore not be optimal as it includes both the innate and acclimated thermal tolerance, which from this study appear to represent two separate biological traits. After the first trial, and the resulting increase in thermal tolerance due to acclimation, the subsequent individual CT_max_ temperatures (trials two to four) were more consistent, which can be seen by the increase in repeatability. The second estimate of repeatability is therefore a more accurate representation of the repeatability of CT_max_ after acclimation. It should be noted however that the sustained “heat hardening” effect observed here may be specific to the species and protocol we used (heating rate of 0.3 min^−1^ & 1 week between trials) as previous experiments have found that heat-shock benefits to thermal tolerance diminish to “pre-hardened” levels after only 24–32 hours^42,44^.

The relatively high level of repeatability in CT_max_ we found is greater than the heritability of thermal tolerance reported in other studies^24–26^, and indeed, the repeatability sets an upper limit for heritability. While it is unclear in the present study how much of the repeatability stems from environmental factors and how much is caused by genetic differences, it does suggest a degree of genetic variation is present in thermal tolerance. Such variation may allow populations to evolve their thermal tolerance, aiding in range expansion and coping with climate change.

The lack of difference in growth between the sham and repeatability groups shows that multiple CT_max_ tests do not impose a growth penalty on the fish. Decreased growth was expected in the repeatability fish compared to the sham fish as the heat stress the fish experience during a CT_max_ trial could trigger an energetically costly stress response, hence diverting energy away from processes such as growth^45^. It is also conceivable that cell and tissue damage could occur during heat shocks that could require costly repair processes. This was not the case however, perhaps because of the short exposure to high temperatures (the duration of the CT_max_ trial was approximately 40 minutes, and the temperature was only high enough to cause agitated behaviour for the final minutes), or because zebrafish are a robust and tolerant species^46–48^. Indeed, the fish regained equilibrium within seconds of returning to 28 °C, and would resume feeding within minutes when presented with food. Similar growth rates to what we observed in both the sham and repeatability fish have been shown for adult zebrafish^49,50^ suggesting that additional experimental procedures (e.g. tagging and anaesthesia) had no major negative impact on the growth rates we observed here.

Contrary to the general perception that larger individuals have a lower thermal tolerance than smaller individuals^18,51,52^, we found no relationship between CT_max_ and length, weight or condition. Similarly, no effect of length^53^ or weight^54^ on CT_max_ has been reported in other species, suggesting a species-specific effect. A minor effect of size on thermal tolerance in these zebrafish may have gone undetected due to a limited size range in the current experiment.

Additionally, CT_max_ did not differ between the sexes, which might be important in order to maintain a balanced sex ratio in populations facing heat spell challenges.

In summary, we have shown that CT_max_ increases after an initial heat shock, which, in turn increases the repeatability of the trait within individuals in subsequent trials. In addition, individuals with a low innate thermal tolerance have a greater acclimation response after heat shock than individuals with a higher innate thermal tolerance. A repeatability estimate of 0.45 (0.28–0.65) after acclimation shows that CT_max_ is a repeatable, and therefore useful measure of thermal tolerance. However, a reliable estimate of repeatability of innate CT_max_ was not possible to achieve, as fish are only naïve to heat shock at the first trial. Furthermore, no growth penalty was imposed on zebrafish after repeated CT_max_ measurements. This suggests the method used does not have major negative physiological impacts on zebrafish, further validating it as a method and valuable metric for continued use within thermal biology.

## Materials and Methods

The experiments were conducted in July 2016 using ornamental zebrafish (Tropehagen Zoo, Trondheim, Norway), which were housed in the animal facility at the Norwegian University of Science and Technology for four months under controlled conditions prior to the experiment. At the start of the experiment, 78 adult zebrafish were tagged (see below) and distributed randomly into four 63-L glass aquaria housing tanks: two sham tanks and two repeatability tanks, all with a maximum density of 4 fish/10 L. The temperature of the housing tanks was kept at 28 °C and the water was well aerated. Each tank was fed 0.1 g of TetraMin dry flakes four times a day, and live Artemia was provided once every two days, replacing one of the TetraMin feeds. The fish were fasted for 20–28 hours prior to critical thermal maxima tests.

The experiments were approved by the Norwegian Animal Research Authority (Permit Number: 8578) and all methods were performed in accordance with the relevant guidelines and regulations.

### Experimental design

To estimate the repeatability of CT_max_, 78 fish were randomly assigned to the control group or the repeatability group. In the repeatability group, each individual fish underwent a CT_max_ test four times with a week between each trial: 7^th^, 14^th^, 21^st^ and 28^th^ of July 2016. To control for any physiological consequences of carrying out CT_max_ experiments 38 fish were assigned to the sham group. These fish underwent a sham CT_max_ test that was identical to the real CT_max_ tests but with water kept constant at 28 °C throughout the first three trials before undergoing an actual CT_max_ test in the fourth trial. Length (to the nearest 0.1 mm) and weight (to the nearest 0.01 g) were measured for all fish after each trial, which allowed for growth comparisons between the sham and repeatability groups. By undergoing an actual CT_max_ test during the fourth trial, handling stress and measurement error were controlled for by allowing comparisons to be made between the CT_max_ of the sham fish with the first CT_max_ trial of the repeatability group. Trials were carried out on the same days for both the sham and the repeatability fish.

### Tagging

All fish were tagged with visible implant elastomer (VIE) tags (Northwest Marine Technology, Shaw Island, WA, USA) allowing for individual identification throughout the experiment. Prior to tagging, the fish were anaesthetized in 110 mg/L buffered tricaine methane sulfonate (MS222). Tags were injected at two of three locations: the base of the dorsal fin, anal fin and caudal peduncle according to^55^. After tagging the fish were weighed to the nearest 0.01 g and photographed using a standardised setup on millimetre paper for measurement of total length to the nearest 0.1 mm, which was quantified using the line function in the program ImageJ (https://imagej.nih.gov/ij/).

### Critical thermal maxima (CT_max_) test

**Figure 5:**
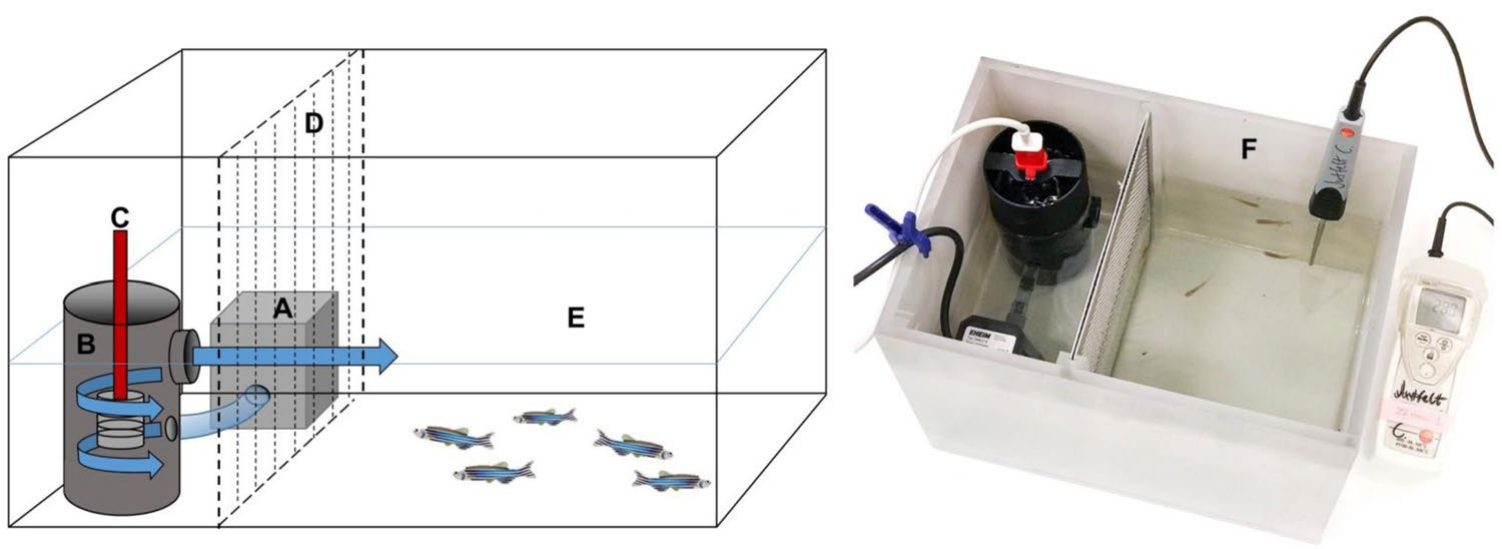
CT_max_ experimental setup, ensuring homogeneous water temperature and consistent heating rate for all trials. (**A**) Water pump; (**B**) custom-made cylindrical steel heating case; (**C**) 300 W coil heater; (**D**) mesh (preventing fish swimming into the heating compartment); (**E**) fish compartment with 9 L of 28 °C water; (**F**) Photograph of the CT_max_ box.

For the CT_max_ test, a heating tank (25 × 22 × 18 cm) was filled with 9 L of 28 °C water. A water pump (Eheim Universal 300, Deizisau, Germany) was attached to a custom-made cylindrical steel heating case consisting of an inflow nipple, a wide outflow and a 300 W coil heater (Fig. 5). The pump pushed water through the heating cylinder and into the fish arena creating stirring, and the heating system was separated from the fish arena by a mesh. This setup ensured a homogenous temperature in the entire arena (<0.1 °C), whilst minimising the water current within the tank. A recently factory calibrated high precision digital thermometer with a ±0.1 °C accuracy (testo-112, Testo, Lenzkirch, Germany) continuously measured the water temperature in the fish compartment.

A group of randomly selected individuals (3–6) were caught from their holding tank and transferred to the heating tank. The water was then heated at a steady rate of 0.3 °C per minute^11^, in accordance with^17^. A pilot experiment with zebrafish instrumented with small thermocouples showed that this heating rate caused a lag of heating of less than 0.2 °C between the ambient water temperature and the deep dorsal muscle (see below). In addition, oxygen concentrations at or above 100% saturation were retained throughout the test. Loss of equilibrium (LOE), defined as uncontrolled and disorganised swimming for two seconds, was chosen as the CT_max_ endpoint^56^. Once LOE occurred in an individual, the water temperature was recorded with an accuracy of 0.1 °C, and the fish was immediately transferred into an individual tank of 28 °C water for recovery. Once the fish had recovered, it was anaesthetised, identified, weighed and photographed before being returned to its holding tank. Recovery of equilibrium generally occurred within two minutes, and normal behaviour was restored after approximately five minutes. During pilot experiments fish commenced feeding within fifteen minutes of a completed CT_max_ test, indicating that the thermal challenge didn’t cause major trauma. All but one of the fish recovered after the CT_max_ tests in the experiment (99% survival). Additionally, two treatment fish do not have a fourth CT_max_ measurement as they jumped out of the heating tank during the test.

The sham CT_max_ test consisted of the fish being put into the heating tank for the same duration (~40 minutes) as the treatment fish but the water was kept at 28 °C throughout. At the end, the fish were individually removed, anaesthetised, weighed, photographed and returned to their holding tank.

A condition index (K, equation 1) was calculated for each fish using the total length and weight measurements. Specific growth rate (SGR, equation 2) was calculated for four growth intervals, the first one from the date of first tagging until the first CT_max_ test, and weekly thereafter.

1 $$K=(Weight/Lengt{h}^{3})\times 100$$

2 $$SGR=(ln({W}_{t})-ln({W}_{0})/time\,interval)\times 100$$

### Thermal ramping rate and muscle temperature

To investigate whether there was a lag between body temperature and water temperature at a thermal ramping rate of 0.3 °C min^−1^ a pilot experiment was carried out. Two zebrafish were anaesthetised in 110 mg/L buffered tricane methane sulfonate (MS-222) before a small thermocouple was inserted into the dorsal muscle to a depth of approximately 2 mm on each fish so the tip of the thermocouple was not visible under the skin and deep enough so the thermocouple held in place. The fish were then carefully placed in the CT_max_ heating tank with MS-222 at a concentration of 55 mg/L in the water to keep the fish anaesthetised throughout the procedure. Another thermocouple was placed in the water to measure ambient water temperature. All thermocouples were calibrated before the experiment.

**Figure 6:**
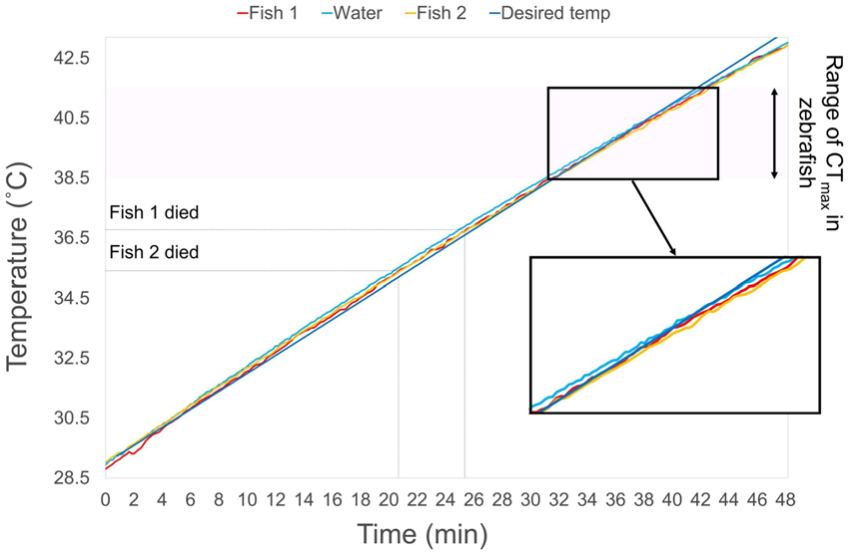
Body temperature of deep dorsal muscle of two zebrafish (Fish 1, weight = 0.4 g, length = 28 mm: red line; Fish 2, weight = 0.4 g, length = 26 mm: yellow line) plotted against ambient water temperature (light blue line) shows a lag in temperatures of less than 0.2 °C. The desired thermal ramping rate of 0.3 °C min^−1^ (dark blue line) is also plotted to show that the ambient water temperature closely follows the desired water temperature.

The CT_max_ method was then carried out using the same heating rate described above and as shown in the Fig. 5. Temperatures were recorded every 10 seconds from both the thermocouples in the fish and the ambient water and the temperature was ramped until reaching 43 °C. The operculum movement of the fish was monitored during the process and the temperature at which the fish ceased breathing was recorded. There was a lag of less than 0.2 °C between the body temperature of the fish and that of the water temperature and no obvious difference in temperature lag was observed when the fish were alive and after they died (Fig. 6).

### Statistical analyses

All statistical analyses were conducted in R 3.3.0 (R Core Team, 2016) with effect sizes with p-values less than 0.05 considered statistically significant. The repeatability of CT_max_ and the corresponding 95% confidence intervals were estimated with generalised linear mixed effect model’s (GLMM’s) and a Bayesian approach using the function MCMCglmm()^57^ and coda’s HDPinterval() function^58^ based on the method recommended by Dingemanse & Dochtermann^59^. Individual identity was included as a random factor, and week number was included as a fixed effect in the model to account for any variation caused by the order of the measurements. Two repeatability measures were estimated, the first using all trials and the second omitting trial 1. The latter was estimated to determine whether the effect of the first thermal challenge had a long-lasting effect on the fish’s CT_max_ in subsequent trials.

A linear mixed effect (LME) model was used to test whether CT_max_ changed between trials, using the lmer() function within the lmerTest package^60^. Individual identity was included as a random factor to account for multiple measures of the same individuals.

An individual’s acclimation response was calculated by subtracting their CT_max_ in trial 1 from their CT_max_ in trial 2 and a linear regression model was used to test the relationship between the first CT_max_ (trial 1) and this acclimation response. Sex was also included in the model, as well as the interaction between sex and the first CT_max_. The correlation between CT_max_ in trial 1 and trial 2 was also tested using Pearson’s product-moment correlation coefficient.

Growth (SGR), sex and treatment (repeated or sham) were analysed using a two-way analysis of variance (ANOVA). SGR is given as the mean for each sex ± standard error of mean (s.e.m.).

Linear regressions were used to test the effect(s) of length, weight and condition (K) on CT_max_. Length, weight and condition were all tested in separate models due to the significant positive correlation between them (Length & Weight, r = 0.76, p < 0.001; Length & Condition, r = 0.28, p < 0.001; Weight & Condition, r = 0.83, p < 0.001). Due to these correlations, weight was chosen as an appropriate proxy for size. A linear mixed effect (LME) model was used to test whether CT_max_ differed between the sexes, or changed with weight (covariate). As weight could have differed between sexes, an interaction was included for weight and sex and the model accounted for repeated measures with individual identity as a random factor.

### Data availability

The dataset generated during the current study is available in the figshare data repository (doi:10.6084/m9.figshare.6148373).
